# *Brucella ceti* and *Brucella pinnipedialis* genome characterization unveil genetic features that highlight their zoonotic potential

**DOI:** 10.1002/mbo3.1329

**Published:** 2022-10-26

**Authors:** Massimiliano Orsini, Andrea Ianni, Luca Zinzula

**Affiliations:** ^1^ Istituto Zooprofilattico Sperimentale delle Venezie, Laboratory of Microbial Ecology and Genomics Legnaro Italy; ^2^ Research Unit in Hygiene, Statistics and Public Health Campus Bio‐Medico di Roma University Rome Italy; ^3^ Department of Molecular Structural Biology Max Planck Institute of Biochemistry Martinsried Germany; ^4^ Centro di Educazione Ambientale e alla Sostenibilità (CEAS) Laguna di Nora Pula Italy

**Keywords:** *Brucella ceti*, *Brucella pinnipedialis*, brucellosis, genome annotation, marine mammals, virulence factors

## Abstract

The Gram‐negative bacteria *Brucella ceti* and *Brucella pinnipedialis* circulate in marine environments primarily infecting marine mammals, where they cause an often‐fatal disease named brucellosis. The increase of brucellosis among several species of cetaceans and pinnipeds, together with the report of sporadic human infections, raises concerns about the zoonotic potential of these pathogens on a large scale and may pose a threat to coastal communities worldwide. Therefore, the characterization of the *B. ceti* and *B. pinnipedialis* genetic features is a priority to better understand the pathological factors that may impact global health. Moreover, an in‐depth functional analysis of the *B. ceti* and *B. pinnipedialis* genome in the context of virulence and pathogenesis was not undertaken so far. Within this picture, here we present the comparative whole‐genome characterization of all *B. ceti* and *B. pinnipedialis* genomes available in public resources, uncovering a collection of genetic tools possessed by these aquatic bacterial species compared to their zoonotic terrestrial relatives. We show that *B. ceti* and *B. pinnipedialis* genomes display a wide host‐range infection capability and a polyphyletic phylogeny within the genus, showing a genomic structure that fits the canonical definition of closeness. Functional genome annotation led to identifying genes related to several pathways involved in mechanisms of infection, others conferring pan‐susceptibility to antimicrobials and a set of virulence genes that highlight the similarity of *B. ceti* and *B. pinnipedialis* genotypes to those of *Brucella* spp. displaying human‐infecting phenotypes.

## Graphical abstract

Orsini and colleagues present the whole‐genome characterization of *Brucella ceti* and *Brucella pinnipedialis*, two Gram‐negative bacteria that infect marine mammals causing lethal disease. By de novo assembling of raw sequencing data and comparative annotation of all available genome sequences in databases, the authors provide insights on Brucella genus phylogeny, undertake in‐depth functional analysis of genes involved in virulence and pathogenesis, and highlight the zoonotic potential of these two pathogens that represent a potential threat for the health of coastal communities worldwide.

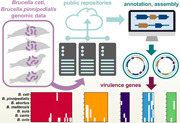

## 1 INTRODUCTION

*Brucella ceti* and *Brucella pinnipedialis* are Gram‐negative bacteria known to infect cetacean and pinniped populations, respectively, and to cause brucellosis—a severe chronic disease—with major clinical signs such as meningoencephalitis, discospondylitis, sub‐cutaneous abscesses, endometritis, and myocarditis, often resulting in affected animals stranding ashore and in fatal outcome (Foster et al., 2007; Guzmán‐Verri et al., 2012; Nymo et al., 2011). As members of the *Brucella* genus within the *Brucellaceae* family, *B. ceti* and *B. pinnipedialis* are closely related to other species that cause brucellosis in terrestrial mammals and, occasionally, in humans (Cloeckaert et al., 2021). However, together with other marine unclassified lineages found in sea otters (Burgess et al., 2017; Miller et al., 2017), they constitute a subgroup that is unique in its ability to live in and spread through the aquatic environment (Guzmán‐Verri et al., 2012; Nymo et al., 2011). Since the first described case in an aborted Bottlenose dolphin (*Tursiops truncatus*) fetus in 1994 (Ewalt et al., 1994), *B. ceti* has been detected among a variety of species from both odontocetes (toothed whales) and mysticetes (baleen whales) groups, including the very recent first isolation from a Risso's dolphin (*Grampus griseus*) and a Killer whale (*Orcinus orca*) (Davison, Dagleish, Dale, et al., 2021), from a Minke whale (*Balaenoptera acutorostrata*) (Davison et al., 2017) and three Sowerby's Beaked whales (*Mesoplodon bidens*) (Davison, Brownlow, et al., 2021). Nevertheless, human cases of *B. ceti* infection have been reported, either as a result of exposure to the pathogen in laboratory settings or naturally acquired and putatively related to raw shellfish consumption (Brew et al., 1999; McDonald et al., 2006; Sohn et al., 2003; Whatmore et al., 2008). Likewise, since its putative first detection in harbor seals (*Phoca vitulina*) in 1994 (Ross et al., 1994), *B. pinnipedialis* was reported to infect a variety of marine mammal species, including both true‐seals (Foster et al., 2018; Hirvelä‐Koski et al., 2017; Kroese et al., 2018; Lambourn et al., 2013) and eared‐ones (Nymo et al., 2018) among pinnipeds, as well as cetaceans of both odontocetes and mysticetes suborders (Buckle et al., 2017; Davison, Dagleish, Ten Doeschate, et al., 2021; Whatmore et al., 2017). The increase of reported *B. ceti* and *B. pinnipedialis* infections in marine mammals, as well as their host range (Foster et al., 2018; Davison, Brownlow, et al., 2021; Davison, Dagleish, Ten Doeschate, et al., 2021; Mauroo et al., 2020) and geographical endemicity expansion (Jensen et al., 2013; Ohishi et al., 2016; Whatmore et al., 2017), are aspects of concern, since they may impact conservation efforts on most vulnerable species already threatened by anthropogenic factors, environmental elements (Van Bressem et al., 2009) or by other pathogens (e.g., morbilliviruses) (Zinzula et al., 2022). Furthermore, the ability of *B. ceti*, and potentially also of *B. pinnipedialis*, to spread across the human population by entering the food chain or after direct contact of humans with infected marine mammals represents a new potential zoonotic threat (Larsen et al., 2018; Maquart et al., 2008; Moreno, 2014; Nymo et al., 2016). Within this picture, undertaking a comprehensive characterization of the *B. ceti* and *B. pinnipedialis* genome is a preliminary and fundamental step to either understand the genetic features that contributed to the evolutionary success of these organisms infecting their hosts and spread through the marine environment or to identify the genetic basis of brucellosis pathological phenotypes. Although research efforts have recently focused in this direction, so far molecular investigations have mainly concentrated on genomic surveillance (Duvnjak et al., 2017; Maquart et al., 2008; Ueno, Kumagai, et al., 2020; Zygmunt et al., 2021) and molecular epidemiology (Garofolo et al., 2020; Kroese et al., 2018; Lambourn et al., 2013; Tian et al., 2019; Ueno, Yanagisawa, et al., 2020), evolutionary phylogenesis (Audic et al., 2011; Duncan et al., 2014; Suárez‐Esquivel et al., 2017; Whatmore et al., 2017) and more recently on the differences in pathogenesis between genotypes (Curtiss et al., 2022; Damiano et al., 2015; Ocampo‐Sosa & García‐Lobo, 2008), whereas functional characterization of *B. ceti* and *B. pinnipedialis* genes has gained less attention. Our study provides a comprehensive description of core‐ and pan‐genome elements from all available (as of February 2022) *B. ceti* and *B. pinnipedialis* full genomes and raw sequencing data, with a specific focus on putative genetic determinants for virulence and pathogenesis. Our findings highlight the zoonotic potential of these emerging pathogens, whose ecological role at the human‐wildlife interface poses a threat to the health of coastal communities worldwide.

## 2 MATERIALS AND METHODS

### 2.1 Available assemblies

The National Center for Biotechnology Information (NCBI) Assembly database (Kitts et al., 2016) was queried for the “*B. ceti*” and “*B. pinnipedialis*” keywords (first access on 23 June 2021, last access on 1 August 2022) and all available assemblies were downloaded (*n* = 15). When accessible, relevant metadata such as date of collection, country of isolation, host species, and pathology were retained.

### 2.2 Raw sequences and assembling

The Sequence Read Archive (SRA) database (Leinonen et al., 2011) was queried with the “*B. ceti*” and “*B. pinnipedialis*” keywords and available experiments were further selected using the SRA Run selector imposing the Illumina platform, and metadata reporting at least the year of isolation and the geographic area. After download, the raw reads were trimmed using the fastp software (Chen et al., 2018), checked for contamination by Confindr software (Low et al., 2019), and only experiments showing a theoretical coverage, calculated on trimmed reads, equal or higher than 70*X*, and no contamination, were retained. Trimmed reads were de novo assembled using the Spades 3.15 software (Bankevich et al., 2012) with default parameters for 2 × 250 or 2 × 150 Illumina paired‐end reads. Scaffolds longer than 200 bp were retained and polished by the Pilon software (Walker et al., 2014).

### 2.3 Criteria for assembly inclusion into data sets

Both the downloaded genomes and those assembled in this study were evaluated by the Quast software (Gurevich et al., 2013), and only those showing an overall size of between 3.0 and 3.4 megabases, the number of contigs <250 and N50 > 125,000 were retained (*n* = 56). As an additional check, assemblies were analyzed to evaluate completeness and contamination by using the CheckM software (Parks et al., 2015), imposing at least 98% of completeness and contamination lower than 2%. Genome assemblies were also checked for the correct species assignment using the Minihash database (Ondov et al., 2016). Finally, the sequence type (ST) was extracted using the multi‐locus sequence typing (MLST) software (Seemann, 2022, https://github.com/tseemann/mlst), and samples missing the call of two or more alleles were discarded. Overall, the criteria for inclusion into the final data sets are summarized in Supporting Information: Table S1: https://doi.org/10.6084/m9.figshare.21251391.v1.

### 2.4 Phylogeny

The phylogenetic placement of *B. ceti* and *B. pinnipedialis* assemblies was performed by alignment‐free single nucleotide polymorphisms (SNPs)‐based approach using the KSNP3 tool (Gardner et al., 2015) to obtain an SNPs matrix, followed by MegaX (Kumar et al., 2018) software analysis for deducing a maximum‐likelihood (ML) phylogenetic tree after choosing the most suitable substitution model as calculated by the Model Selection routine implemented in MegaX. Both downloaded and assembled genome sequences were compared against all the assemblies belonging to the *Brucella* genus at any stage of completeness, retrieved from the NCBI assembly database, as long as they met the above‐mentioned inclusion criteria. The *B. ceti* and *B. pinnipedialis* intra‐genus ML phylogenetic tree was obtained using the RaXML software (Stamatakis, 2014) with default parameters starting from the core genome alignment obtained by the core genome analysis (see below).

### 2.5 Annotation and pan‐genome characterization

The assemblies passing criteria were annotated using a local installation of the Prokka software package (Seemann, 2014) set with default parameters. Pseudogenes were identified using the pseudofinder software (Syberg‐Olsen et al., 2022) starting from the Prokka annotation. The core/pan‐genome investigation was performed by the Panaroo software package (Tonkin‐Hill et al., 2020) with default settings except for the core‐threshold parameter, which regulates the gene detection in the data set for a gene included in the core genome, which was set to 100%. The genome openness was calculated according to the work of Tettelin and colleagues (Tettelin et al., 2008), using the micropan R package (Snipen & Liland, 2015). The pan‐genome protein coding products were annotated according to the clusters of orthologous genes (COG) database (Tatusov et al., 2000) using the NCBI CD‐batch search tool, and the Kyoto Encyclopedia of Genes and Genomes (KEGG) database (Kanehisa et al., 2021) using the KoalaBlast tool at the prokaryotic species level. The pan‐genome was further investigated for its intrinsically pathogenic properties. Briefly, the pan‐genome (including pseudogenes) was compared to a well‐characterized set of protein databases specialized in virulence factors as the virulence factor database (VFDB) (Chen et al., 2005), comprehensive antibiotic resistance database (CARD) (Alcock et al., 2020), Resfinder (Bortolaia et al., 2020), and BacMET (Pal et al., 2014) databases, by the suitable Blast package algorithm (Blastp or tBlastx) (Altschul et al., 1990). The similarity threshold at the protein level and horizontal coverage values were both set to 80% for considering a positive match. Finally, the integrated prophages were searched using the Phage Search Tool Enhanced release (PHASTER) web server (Arndt et al., 2016).

### 2.6 Graphics

Plots were drawn by Prism v.9.3.1 software (GraphPad). Phylogenetic trees were visualized with FigTree v.1.4.4 (http://tree.bio.ed.ac.uk/software/figtree, last accessed on 10 May 2022). Marine mammal icons were retrieved from PhyloPic (credit: Chris huh; http://phylopic.org).

## 3 RESULTS

### 3.1 Characterization of *B. ceti* and *B. pinnipedialis* genomic data set

All *B. ceti* (*n* = 47) and *B. pinnipedialis* (*n* = 9) raw sequencing data available in the NCBI SRA database were downloaded and assembled and, together with already assembled *B. ceti* and *B. pinnipedialis* genomes (nine and six entries, respectively), made up the initial genomic data set for this study. Genome assemblies that did not match our inclusion criteria (see methods) were excluded from further analysis (*n* = 11). Overall, 60 genomic sequences were further characterized, including 50 *B. ceti* (Table 1) and 10 *B. pinnipedialis* genomes (Table 2). Metadata of this initial data set showed that samples were acquired over 26 years (1993–2018) and, when indicated, referred to a large sampling campaign from South America (Costa Rica, *n* = 20), several samplings from Europe (Italy, *n* = 8; Great Britain, *n* = 12; Norway, *n* = 2; Spain, *n* = 3) and one from Asia (Japan, *n* = 1). *B. ceti* samples were collected from specimens belonging to several cetacean species, mostly odontocetes (toothed whales) such as the striped dolphin (*Stenella coeruleoalba*, *n* = 31), the common bottlenose dolphin (*T. truncatus*, *n* = 3), the short‐beaked common dolphin (*Delphinus delphis*, *n* = 3), the harbor porpoise (*Phocoena phocoena*, *n* = 3), and the Atlantic white‐sided dolphin (*Lagenorhynchus acutus*, *n* = 1), but also including mysticetes (baleen whales) such as the common minke whale (*B. acutorostrata*, *n* = 1). Most *B. ceti* samples were isolated from the central nervous system (including the brain, vertebral lesions, and cerebrospinal fluid, *n* = 29) followed by the spleen (*n* = 4), skin or subcutaneous lesions (*n* = 3), lungworms (*n* = 2), lung (*n* = 1), liver (*n* = 1), uterus (*n* = 1), placenta (*n* = 1), and connective tissue (*n* = 1). At the time of collection, signs of neurobrucellosis were reported in most individuals (*n* = 23), one showing signs of focal necrosis in the spleen, the liver, and the lymph nodes, and one displaying osteomyelitis, while in a few others (*n* = 3), no Brucella‐related pathology was observed. Notably, while most of the samples were putatively ascribable to cetacean specimens found either dead or live‐stranded, one was taken from one *T. truncatus* kept in captivity in an aquarium and reported to be affected by osteomyelitis (Table 1). For *B. pinnipedialis*, samples were collected from specimens belonging to the species *P. vitulina* (*n* = 2) and the hooded seal (*Cystophora cristata*) (*n* = 1) among pinnipeds, to *B. acutorostrata* (*n* = 1) among cetaceans, and the Eurasian otter (*Lutra lutra*) (*n* = 1) among other aquatic mammals. Samples were isolated from the spleen (*n* = 3) and lymph nodes (*n* = 2), with one individual showing signs of brucellosis, one with focal necrosis in the liver, and the other two reported as asymptomatic (Table 2). Overall, the size of the genome assemblies in the data set ranged from 3.27 to 3.39 Mb (GC%: 56.09–57.29), while the number of protein‐coding genes returned in the annotation ranged from 3054 to 3251. Furthermore, for all but one sample, the MLST returned an assignment to an already established ST among the ST23, ST26, ST27, and ST24, ST25 for *B. ceti* and *B. pinnipedialis* respectively (Jolley & Maiden, 2010). The remaining sample (*B. pinnipedialis*, GCF_015624465) could not be assigned to any ST due to gaps in one locus (Supporting Information: Table S1: https://doi.org/10.6084/m9.figshare.21251391.v1).

**Table 1:** Data set of *Brucella ceti* full genomes and raw sequencing data

**SRA ID**	**Year**	**Country**	**Host**	**Source**	**Status**
*B. ceti genomes assembled from raw data (this work)*					
ERR418023	2010	Costa Rica	*Stenella coeruleoalba*	Cerebrospinal fluid	Neurobrucellosis
ERR418024	2011	Costa Rica	*S. coeruleoalba*	Cerebrospinal fluid	Neurobrucellosis
ERR418025	2012	Costa Rica	*S. coeruleoalba*	Cerebrospinal fluid	Neurobrucellosis
ERR471312	2007	Costa Rica	*S. coeruleoalba*	Cerebrospinal fluid	Neurobrucellosis
ERR471314^a^	2014	NA	NA	NA	NA
ERR471315^a^	2014	NA	NA	NA	NA
ERR471316	2011	Costa Rica	*S. coeruleoalba*	Cerebrospinal fluid	Neurobrucellosis
ERR471317	2011	Costa Rica	*S. coeruleoalba*	Cerebrospinal fluid	Neurobrucellosis
ERR471318	2012	Costa Rica	*S. coeruleoalba*	Cerebrospinal fluid	Neurobrucellosis
ERR471319	2012	Costa Rica	*S. coeruleoalba*	Cerebrospinal fluid	Neurobrucellosis
ERR471320	2013	Costa Rica	*S. coeruleoalba*	Cerebrospinal fluid	Neurobrucellosis
ERR471321	2013	Costa Rica	*S. coeruleoalba*	Cerebrospinal fluid	Neurobrucellosis
ERR471322	2013	Costa Rica	*S. coeruleoalba*	Cerebrospinal fluid	Neurobrucellosis
ERR471323	2013	Costa Rica	*S. coeruleoalba*	Lung worm	Neurobrucellosis
ERR471324	2013	Costa Rica	*S. coeruleoalba*	Lung	Neurobrucellosis
ERR471325	2012	Spain	*S. coeruleoalba*	Cerebrospinal fluid	Neurobrucellosis
ERR471326	2012	Spain	*Tursiops truncatus*	Vertebral abscess	Neurobrucellosis
ERR471327	2009	Spain	*S. coeruleoalba*	Spleen	Neurobrucellosis
ERR471329	1994	Great Britain	*Delphinus delphis*	Sub‐cutaneous lesion	Asymptomatic
ERR471330	1994	Great Britain	*Phocoena phocoena*	Skin lesion	NA
ERR471333	2005	Great Britain	*S. coeruleoalba*	Brain	NA
ERR473728	2009	Costa Rica	*S. coeruleoalba*	Cerebrospinal fluid	Neurobrucellosis
ERR473729	2011	Costa Rica	*S. coeruleoalba*	Cerebrospinal fluid	Neurobrucellosis
ERR485943^b^	2008	Costa Rica	*S. coeruleoalba*	Cerebrospinal fluid	Neurobrucellosis
ERR485944	2009	Costa Rica	*S. coeruleoalba*	Cerebrospinal fluid	Neurobrucellosis
ERR485945	2009	Costa Rica	*S. coeruleoalba*	Lung worm	Neurobrucellosis
ERR485946	1994	Great Britain	*D. delphis*	Sub‐cutaneous lesion	Asymptomatic
ERR485947	2000	Great Britain	*L. acutus*	Spleen	Focal necrosis
ERR485948	2005	Great Britain	*P. phocoena*	Vertebral lesion	NA
ERR485949	1995	Norway	*Balaenoptera acutorostrata*	Spleen, liver	NA
ERR485951	1997	Great Britain	*D. delphis*	Spleen	NA
ERR554829	1994	Great Britain	*P. phocoena*	Uterus	Asymptomatic
ERR775242	NA	NA	NA	NA	NA
ERR775249	NA	NA	NA	NA	NA
ERR775250	2006	Costa Rica	*S. coeruleoalba*	Cerebrospinal fluid	Neurobrucellosis
ERR775251^b^	2007	Costa Rica	*S. coeruleoalba*	Cerebrospinal fluid	Neurobrucellosis
SRR11510450	2018	Italy	*S. coeruleoalba*	CNS	NA
SRR11510451	2017	Italy	*S. coeruleoalba*	CNS	NA
SRR11510452	2017	Italy	*S. coeruleoalba*	CNS	NA
SRR11510453	2017	Italy	*S. coeruleoalba*	CNS	NA
SRR11510454	2014	Italy	*S. coeruleoalba*	CNS	NA
SRR11510455	2013	Italy	*S. coeruleoalba*	CNS	NA
SRR13221976	2017	NA	NA	Placenta	NA
SRR4038979	NA	NA	NA	NA	NA
SRR4038980	NA	NA	NA	NA	NA
SRR4038981	NA	NA	NA	NA	NA
SRR4039008	NA	NA	NA	NA	NA
*B. ceti genomes already assembled (NCBI SRA)*					
GCA_000157835	NA	NA	NA	NA	NA
GCA_000157855	NA	NA	NA	NA	NA
GCA_000158755	NA	NA	NA	NA	NA
GCA_000158775	NA	NA	NA	NA	NA
GCA_000182425	NA	NA	*T. truncatus*	NA	NA
GCA_000590795	NA	Italy	*S. coeruleoalba*	Brain	NA
GCA_000590815^c^	2012	Italy	*S. coeruleoalba*	Brain	NA
GCA_000662035^c^	2013	NA	NA	NA	NA
GCA_014193785	2018	Japan	*T. truncatus*	Connective tissue	Osteomyelitis

Abbreviation: CNS, central nervous system.

^a^ Genomes excluded from the further analysis did not pass the criteria for species assignment.

^b^ Showed high contamination.

^c^ Showed a low level of completeness.

**Table 2:** Data set of *Brucella pinnipedialis* full genomes and raw sequencing data

SRA ID	Year	Country	Host	Source		Status
*B. pinnipedialis genomes assembled from raw data (this work)*						
ERR471331	1993	Great Britain	*Phoca vitulina*	Spleen		Asymptomatic
ERR471328	1994	Great Britain	*P. vitulina*	Spleen		NA
ERR485950	1994	Great Britain	*Lutra lutra*	Internal iliac lymph node		Asymptomatic
ERR471332	2000	Great Britain	*Balaenoptera acutorostrata*	Mesenteric lymph node		Focal necrosis
SRR4038995	NA	NA	NA	NA		NA
SRR4038996^a^	NA	NA	NA	NA		Brucellosis
SRR825216^b^	NA	NA	NA	NA		NA
SRR825217^b^	NA	NA	NA	NA		NA
SRR825218^b^	NA	NA	NA	NA		NA
*B. pinnipedialis genomes already assembled (NCBI SRA)*						
GCF_015624465	2002	Norway	*Carinaria cristata*	Spleen		NA
GCA_000221005	NA	NA	NA	NA		NA
GCA_000740275	NA	NA	NA	NA		NA
GCF_000157815	NA	NA	NA	NA		NA
GCF_000158675	NA	NA	NA	NA		NA
GCF_000157795^b^	2009	NA	NA	NA		NA

^a^ Genomes excluded from the further analysis showed high contamination.

^b^ Showed a low level of completeness.

### 3.2 Phylogenetic placement of *B. ceti* and *B. pinnipedialis* strains

**Figure 1:**
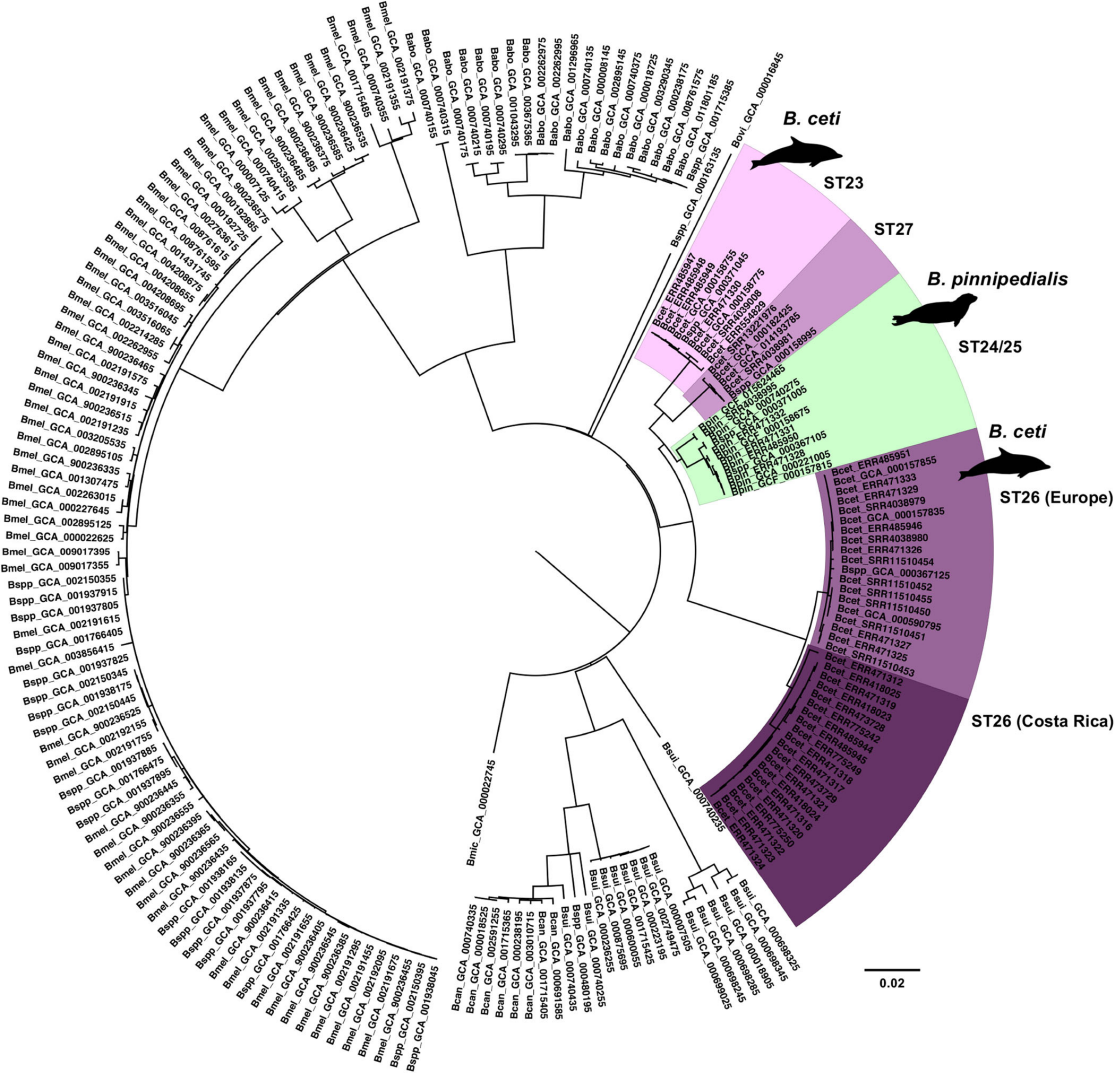
*Brucella ceti* and *Brucella pinnipedialis* intra‐genus phylogenetic placement. Aquatic Brucella (*B. ceti* and *B. pinnipedialis*) cluster in a monophyletic clade close to the one of known species within the genus that are pathogenic in humans. *B. ceti* ST26 genomes from Costa Rica and Europe are highlighted in deep and pale purple, respectively. *B. ceti* ST23 and ST27 are highlighted in pale pink and light magenta, respectively, whereas *B. pinnipedialis* genomes with undefined STs are highlighted in green mint. The tree was deduced based on a total number of 32,755 SNPs. SNP, single nucleotide polymorphism; ST, sequence type.

**Figure 2:**
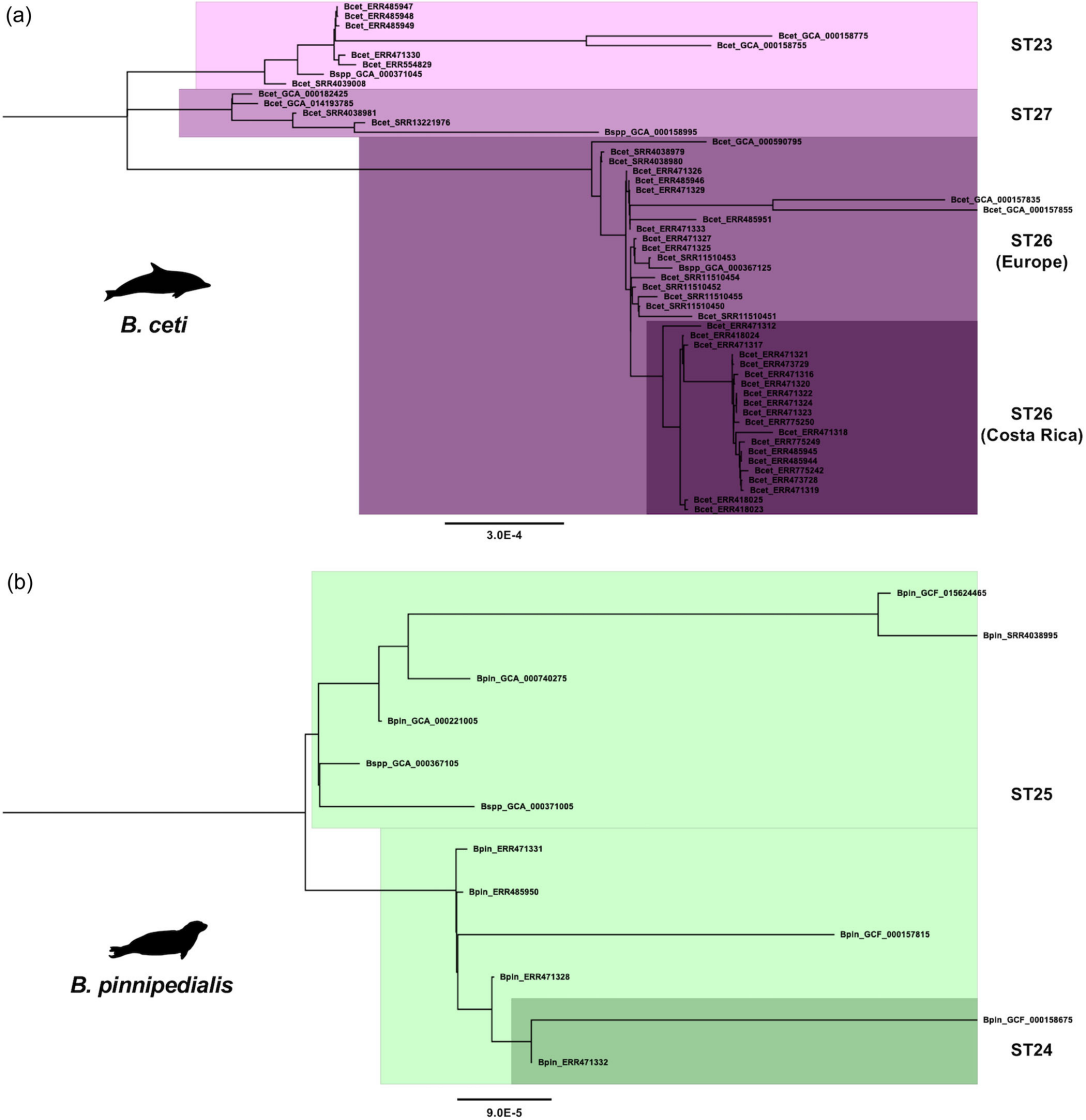
*Brucella ceti* and *Brucella pinnipedialis* intra‐species phylogenetic analysis. (a) *B. ceti* clusters into three clades corresponding to analogous STs, of which ST26 shows a monophyletic structure. ST26 genomes from Costa Rica and Europe are highlighted in deep and pale purple, respectively. ST23 and ST27 genomes are highlighted in pale pink and light magenta, respectively. (b) *B. pinnipedialis* clusters into two clades corresponding to ST24 and ST25, which are highlighted in green laurel and green mint, respectively. ST, sequence type.

We performed a phylogenetic placement of our genome data set against all sequences from members of the *Brucella* genus available in the NCBI assembly database (last access on 1 August 2022; full list in Supporting Information: Table S1: https://doi.org/10.6084/m9.figshare.21251391.v1), that matched inclusion criteria, based on SNPs. Results confirmed what was reported in other studies (Wattam et al., 2009, 2012), that is all the strains belonging to species related to an aquatic environment (i.e., *B. ceti* and *B. pinnipedialis*) group as a single monophyletic clade, clustering at the same level of the *Brucella suis/Brucella canis* group, and of the *Brucella ovis*, *Brucella abortus*, and *Brucella melitensis* clades (Figure 1). All other *Brucella* spp. sequences, including the only genome assembly available for *Brucella microti*, position in early divergent separated clusters (Figure 1). Noteworthily, our phylogenetic analysis revealed that 10 NCBI assemblies previously annotated as *Brucella* spp. (GCA_000371045, GCA_000158995, GCA_000367125, GCA_000371005, and GCA_000367105) unequivocally cluster within the aquatic brucellae monophyletic clade, thereby expanding the current knowledge about the landscape of genomic diversity among those species. These were coming from Europe (Great Britain, *n* = 2) and North America (the United States, *n* = 1) and were sampled from *P. vitulina* (*n* = 3) and *L. acutus* (*n* = 1). We, therefore, included these samples in the following analysis raising our final data set to 53 *B. ceti* and 12 *B. pinnipedialis* genomes, respectively (Table 3). Similar results were observed for several other assemblies generically annotated as *B. spp*, which ended up clustering in well‐defined terrestrial species clades (Figure 1). Moreover, in the monophyletic clade of the aquatic brucellae, *B. ceti* samples form a paraphyletic subclade, similarly to what was reported by Suárez‐Esquivel and colleagues in their multi‐locus variant analysis (MLVA)‐based analysis (Suárez‐Esquivel et al., 2017). Indeed, the *B. ceti* subclade shows a bipartite structure, where a large, compact group includes all ST26 samples, whereas a minor group is further divided into two sister *B. ceti* subgroups, gathering all ST23 and ST27 samples. Altogether the ST23 and ST27 *B. ceti* sister subgroups cluster as a sister group of the *B. pinnipedialis* (Figure 1). Furthermore, ML analysis of *B. ceti* and *B. pinnipedialis* core genome‐derived alignments allowed us to better sort out the relationship between samples. Interestingly, the *B. ceti* phylogeny confirmed the ST‐related structure, except for the ST26. While this group showed a bipartite structure in the SNP‐based analysis, it emerged from the ML analysis as a monophyletic group comprising both European and Costa Rica‐related samples (Figure 2a). Likewise, the *B. pinnipedialis* SNP‐based phylogeny showed the gathering of samples from different STs to the same clade and subclades (Figure 1). Conversely, the core genome ML‐base analysis revealed clustering of the samples according to the ST, grouping ST24 and ST25 in two different subclades (Figure 2b).

**Table 3:** Data set of *Brucella* spp full genomes assigned to *Brucella ceti* and *Brucella pinnipedialis*

SRA ID	Year	Country	Host	Source	Status
*B. ceti genomes already assembled (NCBI SRA)*					
GCA_000371045	1999	USA	*Phoca vitulina*	NA	NA
GCA_000158995	2001	Great Britain	NA	NA	NA
GCA_000367125	1997	NA	*Lagenorhynchus acutus*	NA	NA
*B. pinnipedialis genomes already assembled (NCBI SRA)*					
GCA_000371005	NA	NA	*P. vitulina*	NA	NA
GCA_000367105	2005	Great Britain	*P. vitulina*	NA	NA

### 3.3 Characterization of *B. ceti* and *B. pinnipedialis* pan‐genome

**Figure 3:**
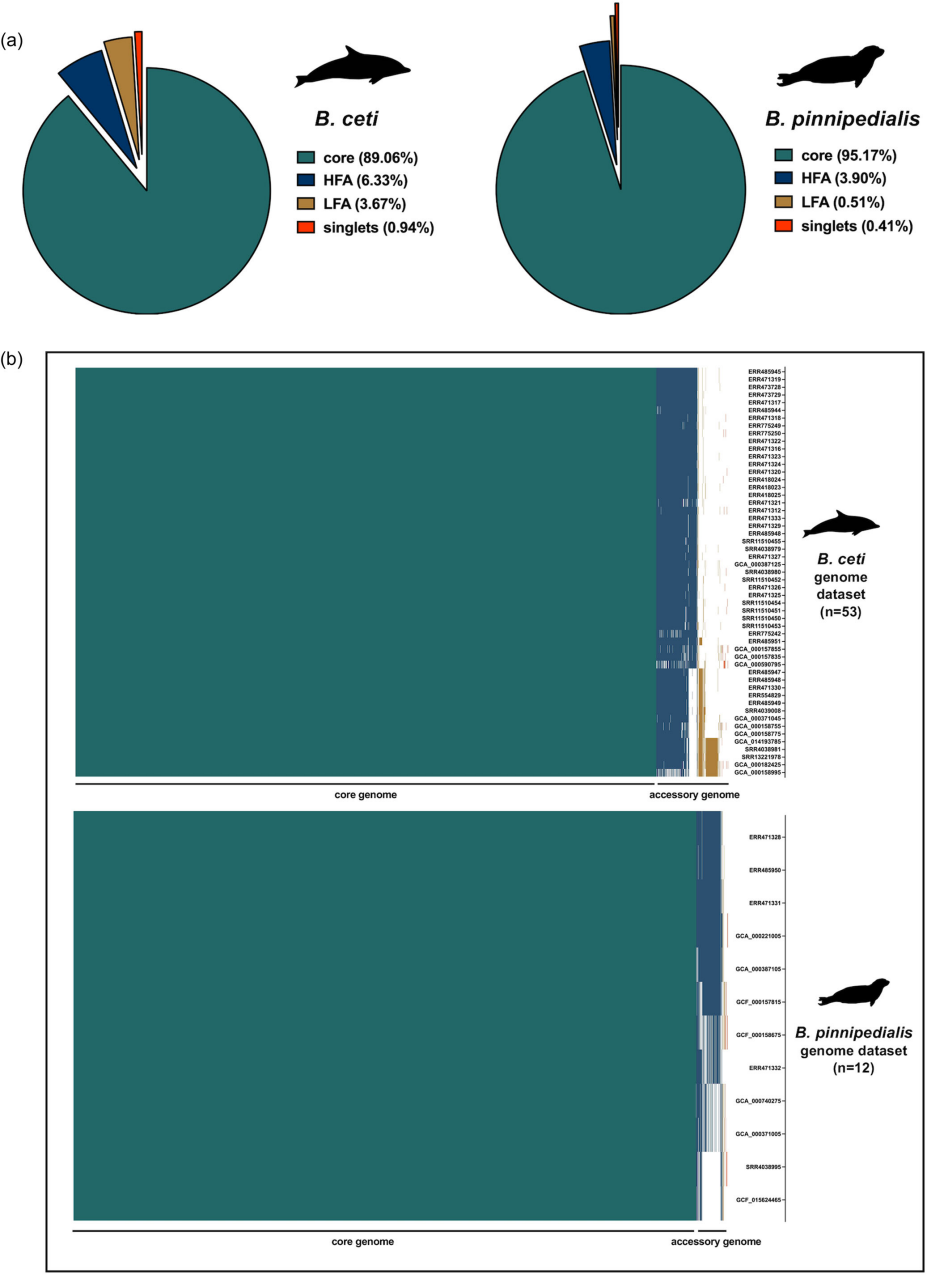
Characterization of *Brucella ceti* and *Brucella pinnipedialis* pan‐genome. (a) Relative composition of *B. ceti* (left panel) and *B. pinnipedialis* (right panel) pan‐genome into core, HFA, LFA, and singlets gene categories. (b) Distribution of *B. ceti* (upper panel) and *B. pinnipedialis* (lower panel) gene categories within the genomic data set. HFA, high‐frequency accessory; LFA, low‐frequency accessory.

Annotation of the 53 *B. ceti* genomic samples resulted in a pan‐genome composition of 3192 loci, of which 2841 (∼89%) are located in the strict‐core genome (namely, 100% of the data set as a more conservative criterion instead of the canonical 95% commonly adopted for the core genome definition) (Brockhurst et al., 2019), 319 (∼10%) are in the accessory genome, and 32 (∼1%) are singlet genes annotated only in one sample each (Figure 3a). Since we adopted a conservative approach to defining the core genome, the accessory one was further divided into high‐frequency accessory (HFA) and low‐frequency accessory (LFA) genes (according to their presence in more or less than 50% of the data set), the two distinct groups accounting for 202 and 117 loci, respectively (Figure 3a). Among them, three sets of genes characterizing the different *B. ceti* STs emerged. One set of 37 genes in the HFA subcategory, absent from those in ST23 and ST27, is unique to the ST26 samples (Figure 3b). These were mainly identified as uncharacterized proteins, with an exception made for some encoding for known proteins including the flgJ peptidoglycan hydrolase, the elastin, the virulence‐associated protein E, and a Toll‐interleukin‐1 receptor (TIR) domain‐containing protein (Supporting Information: Table S2: https://doi.org/10.6084/m9.figshare.21251394.v1). Another set of 20 genes, observed in the LFA subcategory, is unique to the ST23 and ST27 samples and consists of several uncharacterized proteins and some with known functions, such as ATP‐binding cassette (ABC) transporters, extracellular solute‐binding proteins and an endoribonuclease (Figure 3b). Finally, a set of 63 genes, observed in the ST27 samples only and, in addition to some uncharacterized proteins comprises ABC transporters, transposases, DNA binding and metabolism enzymes, regulators of transcription, amino‐acids metabolism enzymes, peptidoglycan binding proteins, one porine and, noteworthy, one type II anti‐toxin protein and one multidrug resistance efflux transporter family protein (Figure 3b and Supporting Information: Table S2: https://doi.org/10.6084/m9.figshare.21251394.v1). The annotation of the 12 *B. pinnipedialis* genomic samples resulted in a pan‐genome composition of 3151 loci, with a core genome of 2998 genes (∼95%), an accessory genome of 139 genes (∼4%), and just 13 singlets (∼0.4%) (Figure 3a). HFA and LFA genes accounted for ∼3.9% and ∼0.5% of the data set, respectively. Furthermore, no specific sets of genes in the accessory genome could be uniquely associated with any of the two ST24 and ST25 samples (Figure 3b). Among singlets of *B. ceti* (*n* = 32) and *B. pinnipedialis* (*n* = 12), 10 and 4 genes were found encoding for known proteins, respectively, whereas the remaining ones were either associated with hypothetical proteins or did not have any match within the NCBI *nr* database (Supporting Information: Table S2: https://doi.org/10.6084/m9.figshare.21251394.v1). Singlet genes from both species were excluded from subsequent analysis.

### 3.4 *B. ceti* and *B. pinnipedialis* comparative pan‐genome model

**Figure 4:**
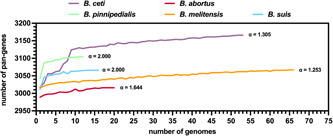
*Brucella ceti* and *Brucella pinnipedialis* pan‐genome model. Comparative analysis of the *B. ceti* and *B. pinnipedialis* pan‐genome profile against that of zoonotic species *B. abortus*, *B. melitensis*, and *Brucella suis*; alpha values calculated according to the Heaps law are indicated.

Comparative analysis of the total genes and conserved ones as a function of the genome number of aquatic *B. ceti* and *B. pinnipedialis*, as well as of terrestrial *B. abortus*, *B. melitensis*, and *B. suis* genomes, clearly showed that all species have a close genome (Figure 4). As shown by the alpha values calculated according to the Heaps law described by Tettelin and colleagues (Tettelin et al., 2008), *B. abortus*, *B. melitensis*, and *B. suis* genomes reach their plateau in the 3016–3067 pan‐gene range, thereby featuring the criteria for closeness definition (Keller & Ankenbrand, 2021) (*α*_*B.abortus*_ = 1.644; *α*_*B*__.__*melitensis*_ = 1.253; *α*_*B.suis*_ = 2.000). Assignment of closeness to the *B. ceti* genome, which contains up to 3166 pan‐genes, is the result of an alpha value that lies in between those of *B. abortus* and *B. melitensis* (*α*_*B.ceti*_ = 1.305). Yet, the *B. ceti* genome displays a clear step‐wise trend, opening the possibility that it may be prone to horizontal gene transfer events (Figure 4). Possibly, such difference in the genome profile trend of *B. ceti* can be ascribed to the contribution brought by samples from the ST23 and ST27 groups of the data set, which are enough genetically divergent from each other and distant from ST26 samples (Figure 3b). *B. pinnipedialis*, whose genome possesses 3105 pan‐genes, displays an alpha value identical to that of *B. suis* (*α*_*B.pinnipedialis*_ = 2.000) and a profile of continuous trend, which together clearly support genome closeness assignment (Figure 4). However, given that the number of available *B. ceti* and *B. pinnipedialis* genomic sequences is still limited, a statement about the closeness of their genome could still be susceptible to revision in case more sequencing data become available.

### 3.5 Functional annotation of *B. ceti* and *B. pinnipedialis* pan‐genome

**Figure 5:**
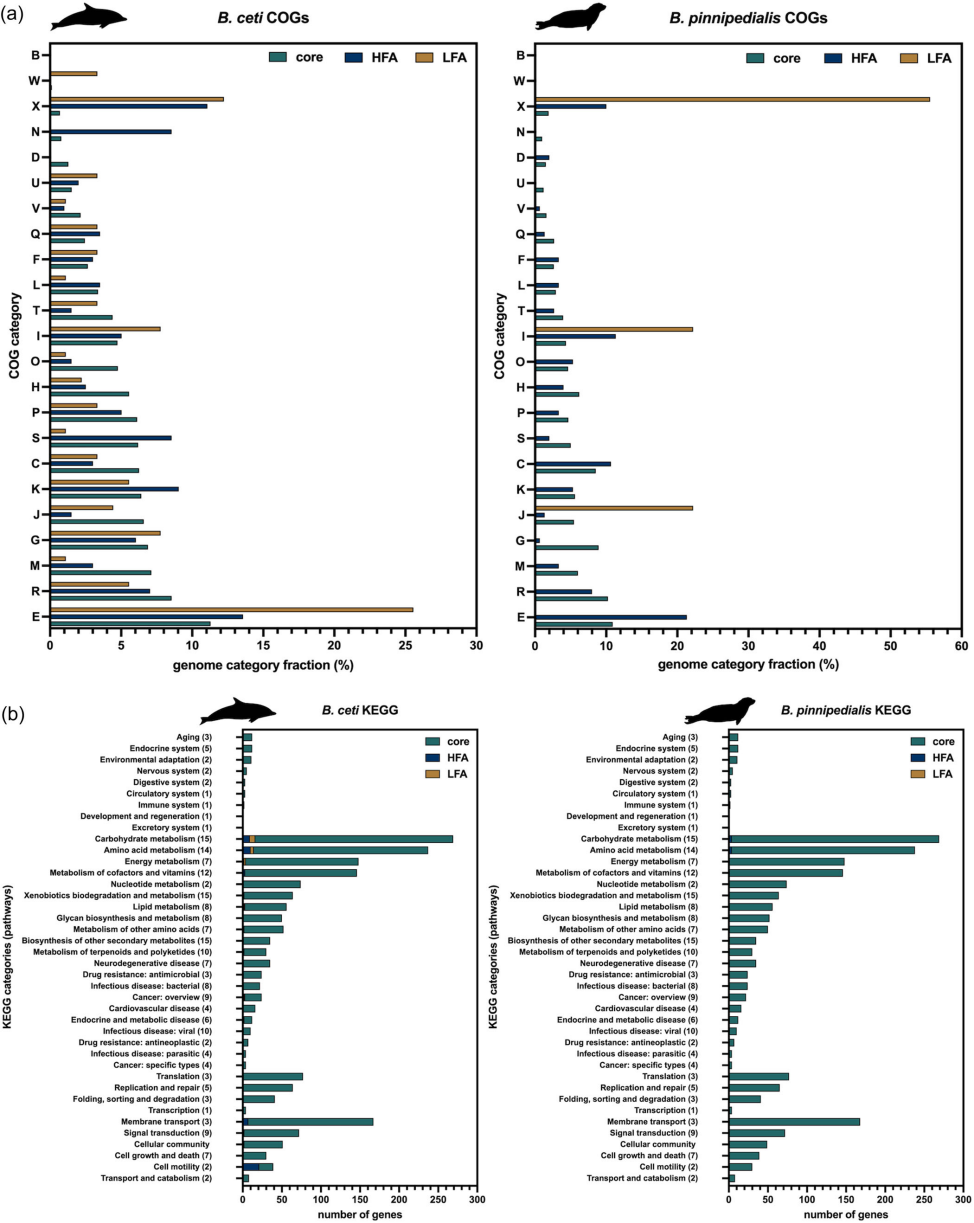
COG categories and KEGG pathways in the *Brucella ceti* and *Brucella pinnipedialis* pan‐genome. (a) Relative distribution of *B. ceti* (left panel) and *B. pinnipedialis* (right panel) core and accessory genes within the data set assigned to different COG categories. (b) Relative distribution of *B. ceti* (left panel) and *B. pinnipedialis* (right panel) core and accessory genes within the data set assigned to different KEGG pathways. COG, clusters of orthologous genes; KEGG, Kyoto Encyclopedia of Genes and Genomes.

Among *B. ceti* gene products, 2490 out of 2841 (87.6%) from the core genome, and 198 out of 319 (62.1%) from the accessory one, were annotated in the least one category of the COG database (Tatusov et al., 2000) (Figure 5a). Moreover, gene products belonging to multiple COG categories were 170 from the core genome and 22 from the accessory one (17 HFA and 5 LFA genes, respectively). Functional protein domains among *B. ceti* gene products were most frequently annotated as belonging to the COG category E (amino acid metabolism and transport), accounting for 11.8% of all annotated domains. Other frequently represented COGs were the R (general functional prediction only), M (cell wall/membrane/envelope biogenesis), G (carbohydrate metabolism and transport), J (translation), K (transcription), C (energy production and conversion), P (inorganic ion transport and metabolism), and the S (function unknown) categories. On average, each of these accounted for more than 6% of the core genome and 1.1%–9.0% of the accessory one (Figure 5a). Other COGs followed, each accounting for 0.03%–5.29% of the total annotated domains. Some of them were almost equally distributed in the three genome categories, such as the F (nucleotide transport and metabolism) and the Q (secondary metabolite biosynthesis transport and catabolism), whereas COGs such as the L (replication recombination and repair), X (mobilome: prophages, transposons), N (cell motility), and W (extracellular structures) were represented by genes located almost exclusively in the accessory genome (Figure 5a). Annotation of the *B. pinnipedialis* pan‐genome showed a similar distribution among the most frequent COG categories, with 2606 out of 2998 (86.9%) annotated gene products from the core genome, and 73 out of 139 (52.5%) from the accessory one. However, some differences with respect to the *B. ceti* genome were observed. COG categories T (signal transduction mechanisms), V (defense mechanisms), U (intracellular trafficking, secretion, and vesicular transport), W, D (cell cycle control), and B (chromatin structure and dynamics) were exclusively represented by gene products from the core genome, the X was represented only by genes located in the accessory genome and the N was represented by genes equally distributed between the core and the accessory genome (Figure 5a). Furthermore, analysis of the *B. ceti* pan‐genome based on the KEGG database (Kanehisa et al., 2021) returned 1953 annotated proteins (61.2% of the submitted genes products) spread over 238 pathways, grouped in 40 categories and 66 complete modules (Figure 5b and Supporting Information: Table S3: https://doi.org/10.6084/m9.figshare.21251403.v1). Following a knowledge‐based approach, the further analysis focused on pathways involved in bacterial virulence and pathogenesis. Among the annotated gene products, 40 were assigned to the quorum sensing pathway (KEGG 02024), 11 to biofilm formation pathways (KEGG 05111, 02025, and 02026), 7 to the chemotaxis pathway (KEGG 02030), and 32 to the flagellar assembly pathway (KEGG 02040). Noteworthily, *B. ceti* annotations included products encoded by genes belonging to the type III secretion system (T3SS) FliE‐R and Flh operons, the FlgA‐L operon that encodes for the rod, the P/L ring, and the hook parts of the flagellar machinery as well as products encoded by genes of the hook filament junction FlgK and FlgL operons, the filament part‐coding FliC gene, the MotA‐C operon and the FliY regulator. Overall, 155 gene products were involved in pathways related to the Human Disease KEGG category, of which 22 were specifically linked to bacterial infectious disease pathways (KEGG 05120, 05130, 05131, 05132, 05132, 05133, 05134, 05150, 05152). Other genes, whose annotation points to more generic human pathways (e.g., Cardiovascular Disease or Cancer) were not taken into consideration, since their annotation process can be influenced by higher hierarchical categorization. Moreover, 24 gene products were associated with patterns for antimicrobial drug resistance, including 11 of the beta‐lactam (KEGG 015), 5 of the vancomycin‐resistance (KEGG 01502), and 8 of the cationic‐antimicrobial (KEGG 01503) pathways. Nevertheless, none of the five gene products related to the vancomycin‐resistance pathway is encoded by genes belonging to the Van operon (Courvalin, 2006), which may be indicative of a non‐fully functioning related pathway. Similarly, while efflux pumps were among the gene products related to the beta‐lactam resistance pathway (Gruenheid & Moual, 2012), the lack of products encoded by genes such as BlaI/Z and AmpR/C suggests that the metabolic pathway branch leading to the beta‐lactam degradation is somehow interrupted. In addition, no gene products were assigned to any heavy‐metal resistance pathway, with the only exception of three gene products putatively involved in conferring resistance to platinum (Supporting Information: Table S3: https://doi.org/10.6084/m9.figshare.21251403.v1). Furthermore, considering the peculiarity of the *B. ceti* aquatic ecological niche compared to the one of other terrestrial species within the genus, we also tried to investigate the presence of proteins whose functions are important to surviving in aquatic environments. Interestingly, we found a match between the *B. ceti* locus group_825 and a DNA‐binding protein (Uniprot P02345) that was identified in the archaeon *Thermoplasma acidophilum* quinone droplets (Nagy et al., 2019) and suggested to wrap DNA in a histone‐like fashion to stabilize it and prevent its denaturation under extreme environmental conditions. For the pan‐genome of *B. pinnipedialis*, the KEGG‐based analysis returned an annotation for 1950 out of 3151 proteins submitted (61.9%), spread over 238 pathways, grouped in 40 categories and 67 modules (Figure 5b and Supporting Information: Table S4: https://doi.org/10.6084/m9.figshare.21251412.v1). Overall, the KEGG assignment profile was identical to that of *B. ceti* genome annotation, with an exception made for the gene products TrbJ and TrbL‐only‐lacking (two Type IV secretion system proteins) which are missing in the *B. pinnipedialis* assignment to the KEGG 02024 quorum sensing pathway (Figure 5b).

### 3.6 *B. ceti* and *B. pinnipedialis* virulence factors genes

**Figure 6:**
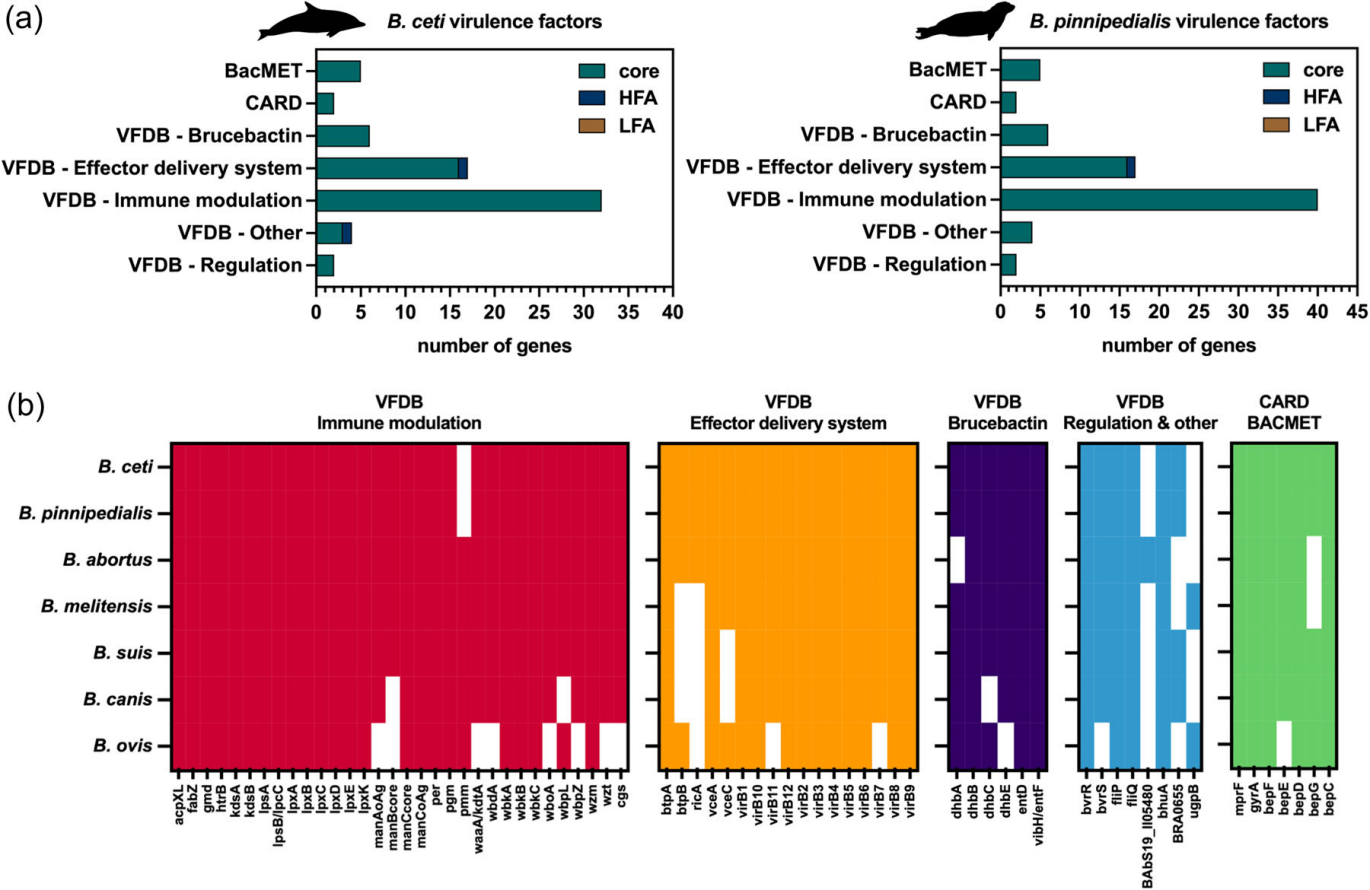
*Brucella ceti* and *Brucella pinnipedialis* virulence factors. (a) Frequency of virulence factors in core and accessory *B. ceti* (left panel) and *B. pinnipedialis* (right panel) genome, as annotated in VFDB, CARD, and BacMET databases. (b) Comparative analysis of *B. ceti* and *B. pinnipedialis* encoded virulence factors against those from genomes of human‐infecting terrestrial zoonotic brucellae *Brucella abortus*, *Brucella canis*, *Brucella melitensis*, and *Brucella suis* species; the nonhuman‐infecting *Brucella ovis* species was taken as outgroup.

To further characterize the *B. ceti* pan‐genome for the presence of determinants of virulence and pathogenesis, our genomic data set was searched against the VFDB (Chen et al., 2005). Results showed 61 entries matching with genes encoding for well‐established virulence factors identified within multiple bacterial species including those in the *Brucella* genus (Figure 6a and Supporting Information: Table S5: https://doi.org/10.6084/m9.figshare.21251406.v1). Of these, 59 entries correspond to products of genes located in the *B. ceti* core genome, whereas two were products of HFA genes of the *B. ceti* accessory genome. Moreover, grouping the gene products according to the VFDB classification, showed that 32 of those genes are involved in immune modulation, including 31 core gene products related to the lipopolysaccharide (LPS) biosynthesis and the product of the ndvB/cgs gene, a cyclic beta‐1,2‐glucano synthase that interferes with the maturation of the phagosomal membrane, thus preventing the *Brucella* containing vacuole (BCV) to fuse with lysosomes (Arellano‐Reynoso et al., 2005). Noteworthy, *B. ceti* genes involved in LPS biosynthesis (Supporting Information: Table S5: https://doi.org/10.6084/m9.figshare.21251406.v1) displayed a 99%–100% sequence similarity with orthologs identified in *B. melitensis* bv.1, *B. suis* and *B. abortus*. Interestingly, the *pmm* gene, conserved in all other Brucella species and contributing to the LPS synthesis and its functions was not found in the *B. ceti* genome (Figure 6b). Conversely, the loci glmM_1 (manBcore gene) and paralogue glmM_2, both ascribable to the bacterial pmm gene family—which encode for a phospho‐mannomutase involved in the fructose‐to‐mannose conversion for the LPS biosynthesis—were found in all *B. ceti* genomes. In addition, 17 gene products grouped under the effector delivery system (EDS) category and showed a 95%–100% sequence similarity with *B. melitensis* bv.1, and *B. suis* orthologs. Of these, 16 and 1 are encoded by genes respectively found in the core and in the accessory genome. According to their putative function, 13 encode for proteins involved in the VirB type IV Secretion System (T4SS), which are required at late stages in the *Brucella* life cycle and govern the interaction of the BCV with the endoplasmic reticulum (Boschiroli et al., 2002). Two gene products were found in all the samples, namely the vceA, and vceC, which are secreted effectors translocated into macrophages by the *Brucella* T4SS (de Jong et al., 2008). We identified the BtpA and BtpB gene products belonging to the EDS category, both acting as substrates for the VirB T4SS and able to interfere with Toll‐like receptors (TLRs) signaling to mitigate the host inflammatory response (Atluri et al., 2011; Salcedo et al., 2013). Moreover, expression of the VirB T4SS itself is controlled by two gene products found in all samples, namely the bvrS and bvrR (Guzmán‐Verri et al., 2002), both encoded by genes in the *B. ceti* core genome (Figure 6b and Supporting Information: Table S5: https://doi.org/10.6084/m9.figshare.21251406.v1). Furthermore, the ricA gene product was found, whose interaction with the host Rab2 protein affects the BCV maturation, thus decreasing intracellular replication rates and contributing to the evasion of the innate immune response (de Barsy et al., 2011). Interestingly, 6 genes, all located in the *B. ceti* core genome are related to the production of brucebactin, a highly‐conserved siderophore firstly identified in *B. abortus* and putatively acting as an iron transporter in iron‐limiting conditions at the beginning of the stationary growth phase (González Carreró et al., 2002). Four additional gene products, all but one encoded by genes in the *B. ceti* core genome, namely the ugpB, bhuA, flhP, and fliQ, are virulence factor candidates according to the VFDB classification. The latter two display significant sequence similarity with orthologs in *Bartonella bacilliformis*, a Gram‐negative bacterium that causes febrile anemia in humans (Scherer et al., 1993). Noteworthily, analysis of the *B. pinnipedialis* data set returned an identical pattern of virulence factors (Figure 6a,b and Supporting Information: Table S6: https://doi.org/10.6084/m9.figshare.21251409.v1). Finally, a comparative analysis of virulence factors was performed against all available genomes from *B. abortus*, *B. canis*, *B. melitensis*, and *B. suis*. As an example of non‐zoonotic Brucella, we also included in our analysis the virulence factors profile of the only‐available *B. ovis* genome. Results showed that *B. ceti* and *B. pinnipedialis* share the most virulence‐related genes with zoonotic terrestrial species, mainly located in the core genome (Figure 6b). The gene BtpB, whose function is to inhibit the innate inflammatory response in alveolar macrophages through the TLR/nuclear factor kappa B (NF‐kB) pathway, and to suppress the formation of cellular reactive oxygen species (ROS) during Brucella infection (Li et al., 2022), is shared by *B. ceti* and *B. pinnipedialis* with *B. abortus* and *B. ovis* (Figure 6b). The gene RicA, which interacts with human Rab2 (de Barsy et al., 2011) and is also required by *B. abortus* for intracellular proliferation (Fugier et al., 2009), is exclusively shared by *B. ceti* and *B. pinnipedialis* with this terrestrial zoonotic Brucella (Figure 6b). Notably, despite all Brucella species being described as nonmotile organisms (Coloma‐Rivero et al., 2021), we detected in all species the two genes flip and fliQ, type III secretion exporters belonging to the flagella pathway. Indeed, since flagellum is known to contribute to virulence, cell growth, and biofilm formation, it is deemed to provide Brucella with peculiar infection versatility (Coloma‐Rivero et al., 2021). In addition, we found that the two genes are closely related to orthologs in species from the genus *Bartonella*, whose members are capable of wide‐range interspecies transmission and long‐lasting bacteremia (Supporting Information: Table S5: https://doi.org/10.6084/m9.figshare.21251406.v1, Supporting Information: Table S6: https://doi.org/10.6084/m9.figshare.21251409.v1). Virulence factors were also searched among pseudogenes (Supporting Information: Table S7: https://doi.org/10.6084/m9.figshare.21251397.v1) to investigate the possible lost role of pseudogenized loci. A total of 47 matches were found against VFDB and variably distributed within the data set. Of these, 10 were detected in all *B. ceti* and *B. pinnipedialis* samples, whereas 15 pseudogenes sporadically occurred each in one sample only. Conversely, some of the remaining matches showed a species‐specific occurrence, with 12 pseudogenes related to virulence patterns observed in *B. ceti* samples only, and one pointing to a glucose/galactose transporter (gluP) exclusively detected in *B. pinnipedialis*. In addition, pseudogenes showed an ST‐specific occurrence, with some putatively encoding for flagellar proteins (flhA, flgG, fliF, and flaA) detected in the *B. ceti* ST26 samples only, and others putatively encoding for effectors of the type IV secretion system, such as the vceA protein, were detected only in the ST27 samples (Supporting Information: Table S7: https://doi.org/10.6084/m9.figshare.21251397.v1).

### 3.7 *B. ceti* and *B. pinnipedialis* antimicrobial resistance (AMR) genes and prophage elements

The presence in our data set of genes encoding for products potentially involved in AMR was assessed by searching against the CARD (Alcock et al., 2020), Bacmet (Pal et al., 2014), and Resfinder (Bortolaia et al., 2020) databases. CARD database returned 2 hits as AMR candidates, which were present in all samples and whose genes are located in the *B. ceti* and *B. pinnipedialis* core genome. These are gyrA, which potentially confers resistance to the nalidixic acid (Khan et al., 2019), and mprF, which encodes for an integral membrane protein that confers resistance to cationic peptides (Ernst et al., 2009) (Supporting Information: Table S5: https://doi.org/10.6084/m9.figshare.21251406.v1, Supporting Information: Table S6: https://doi.org/10.6084/m9.figshare.21251409.v1). Among *B. ceti* gene products, only one match was returned by the Resfinder database, showing 100% identity to the tecC enzyme that confers resistance to tetracycline (Saenger et al., 2000). Notably, this gene product was detected in two samples only, tempting to speculate that *B. ceti* phenotypes may still display a substantial pan‐susceptibility to antibiotics. By contrast, the tecC gene was not detected in any of the *B. pinnipedialis* samples (Supporting Information: Table S5: https://doi.org/10.6084/m9.figshare.21251406.v1, Supporting Information: Table S6: https://doi.org/10.6084/m9.figshare.21251409.v1). Comparison against the Bacmet database returned for both *B. ceti* and *B. pinnipedialis* samples the presence of five genes (namely, bepC, bepD, bepE, bepF, and bepG), all encoding for multi‐drug efflux pumps and involved in the efflux of toxic and hydrophobic compounds, but also contributing to resistance to a drug such as a deoxycholate, sodium dodecyl sulfate and nalidixic acid (Martin et al., 2009; Posadas et al., 2007). Finally, the PHASTER web server (Arndt et al., 2016) was used to identify the *B. ceti* and *B. pinnipedialis* pan‐genome prophage regions which can mobilize genetic determinants (von Wintersdorff et al., 2016). Although a substantial portion of the accessory genome of both species consisted of incomplete‐prophage or questionable‐prophage sequences—possibly representing signals of past infections—results showed no presence of intact prophages (Supporting Information: Table S8: https://doi.org/10.6084/m9.figshare.21251400.v1).

## 4 DISCUSSION

The *Brucella genus* comprehends species such as *B. melitensis*, *B. canis*, *B. abortus*, and *B. suis*, which are all well‐established zoonotic agents, but also species with peculiar host restrictions that ‐ apart from sporadic cases of infection ‐ hamper the spread of these bacteria across the human population (Moreno, 2014). Nevertheless, among these host‐restricted species, the marine mammal‐infecting *B. ceti* and *B. pinnipedialis* are emerging worldwide as pathogens of concern at the human‐wildlife interface of coastal ecosystems (Guzmán‐Verri et al., 2012; Nymo et al., 2011). However, such interface is somehow limited as regards human interactions with marine mammals, and given the overall low prevalence of human brucellosis ascribable to *B. ceti* and *B. pinnipedialis* so far reported, a correct evaluation of the zoonotic potential of these organisms is of utmost importance (Głowacka et al., 2018). A key issue that needs to be addressed is whether the relative rarity of human infections with *B. ceti* and *B. pinnipedialis* is due to low levels of human exposure to the pathogen or rather to the poor ability of these bacteria to infect human hosts. On the one hand, evaluation of the level of exposure is not a trivial task, as it would require large epidemiological studies and extensive surveillance programs (Pereira et al., 2020). So far, most human cases of *B. ceti* infection have been attributed to the assumed consumption of seafood and, apart from cases that involved personnel working in aquatic parks, a clear correlation with a history of direct contact between the patient and infected marine mammals could not be made (Larsen et al., 2018; Maquart et al., 2008; Moreno, 2014; Nymo et al., 2016). On the other hand, thanks to a sufficient number of *B. ceti* and *B. pinnipedialis* genomic sequences sampled and made available in databases, evaluation of the presence of genetic signatures of virulence offers a unique opportunity to decipher the molecular basis for their pathogenesis and to predict their impact on public health. In this work, we have undertaken a comprehensive characterization of all available *B. ceti* and *B. pinnipedialis* genomes. One main limitation of our study is the very few available data when compared to those obtainable for the related zoonotic species within the genus (i.e., *B. melitensis*, *B. canis*, *B. abortus*, and *B. suis*). Only about fifty *B. ceti* and a dozen of *B. pinnipedialis* samples were suitable for the analysis, with half of them coming from a single collection study (Costa Rica, 20 samples), one collected in an aquarium (Japan), and the remaining ones coming from marine mammals stranded mostly along European coasts. Noteworthily, when these samples were phylogenetically compared against all genomic assemblies within the *Brucella* genus, a further group of five samples, previously generically annotated in the NCBI assembly database as *B. spp*, clustered in the aquatic Brucella clade. Therefore. although we were able to infer they belong to both *B. ceti* and *B. pinnipedialis*, these samples are representatives of the existing difficulties in subtyping such organisms. Moreover, albeit informative on the *B. ceti* and *B. pinnipedialis* zoonotic potential, the results obtained in this study cannot be yet representative of the overall population of the two pathogens presently circulating among free‐ranging marine mammals worldwide. Notwithstanding the intrinsic limitations due to the number of samples and their geographic provenance, the genomic data set was however highly valuable in terms of temporal extension of samples collection (spanning over two‐and‐a‐half decades) and the heterogeneity of the infected species, which included several odontocetes and mysticetes among cetaceans, and three ones among caniforms. In this regard, the metadata data set evidenced the absence of host restriction and an overall moderate tissue tropism. A paraphyletic relationship was established between *B. ceti* and *B. pinnipedialis* samples. Furthermore, intra‐genus phylogeny revealed distribution over three *B. ceti* and two *B. pinnipedialis* different STs, with the 20 samples of the subgroup from Costa Rica showing substantial clonality. Analysis of the *B. ceti* and *B. pinnipedialis* pan‐genome revealed the presence of an extended set of pathogenic molecular determinants, including genes for adherence, invasion, survival within host cells, and modulation of the immune response. Moreover, such a wide collection of pathogenic genes is markedly grouped in the core genome. This suggests that *B. ceti* and *B. pinnipedialis* pathogenic genes may account for the intrinsic genetic property of the two species, rather than being genetic features randomly acquired and placed within the accessory portion of their close genome. In addition, molecular determinants of virulence and pathogenesis showed high sequence similarity with their counterparts in *B. abortus*, *B. melitensis*, and *B. suis*/*B. canis*, thus enforcing the notion of *B. ceti* and *B. pinnipedialis* as potential zoonotic pathogens. This evidence is further supported by the fact that, as returned by the whole genome phylogeny analysis, *B. ceti* and *B. pinnipedialis* assemblies fall in a sister clade to that of *B. abortus*, *B. melitensis*, *B. suis*, and *B. canis* genomes. Given that these *Brucella* species were reported as capable of infecting human macrophages and epithelial cells (Larsen et al., 2013), as well as parasites of edible fish such as the Atlantic Cod (*Gauds morhua*) (Larsen et al., 2018; Nymo et al., 2016), investigations towards their zoonotic potential and capability of entering the human food chain should be intensified. Furthermore, we observed that the *B. ceti* and *B. pinnipedialis* genomes are pan‐susceptible to all known antibiotics, and no evidence of recently acquired prophages was detected. Moreover, not all the assembled contigs were ascribable to known plasmids, which suggests that genetic determinants of virulence and pathogenesis cannot in principle be transmitted by conjugation. Nevertheless, we were able to identify antimicrobial genetic determinants in two *B. ceti* genomic samples from the Adriatic Sea, a Mediterranean region that is re‐known for being subjected to high anthropogenic pressure (Basili et al., 2021; Bruschi et al., 2021). These aspects, combined with the genome closeness observed in our *B. ceti* and *B. pinnipedialis* genomic data set, lead to the hypothesis that these bacteria have a natural tendency to remain confined to their particular ecological niche with few opportunities for genomic exchange with other bacterial populations. However, it is tempting to speculate that, when exposed to anthropogenic pressures and high levels of microbial biodiversity, they may readily display capabilities for the lateral acquisition of antimicrobial resistance genes (Wattam et al., 2014). Altogether, these features should act as a warning in arguing that, under the convergence of specific ecological circumstances, *B. ceti* and *B. pinnipedialis* may represent a real threat to human health, thereby improving surveillance on all human activities that can raise the levels of exposure to these organisms a priority in the one‐health agenda.

## AUTHOR CONTRIBUTIONS

**Massimiliano Orsini**: Conceptualization (lead); data curation (lead); formal analysis (lead); investigation (equal); methodology (lead); project administration (lead); supervision (lead); validation (lead); writing–original draft (equal); writing–review & editing (equal). **Andrea Ianni**: Investigation (equal); formal analysis (equal); validation (equal) project administration (equal); writing–review & editing (equal). **Luca Zinzula**: Conceptualization (lead); data curation (lead); formal analysis (equal); investigation (equal); methodology (equal); project administration (equal); supervision (lead); validation (lead); writing–original draft (lead); writing–review & editing (lead).

## CONFLICT OF INTEREST

None declared.

## ETHICS STATEMENT

None required.

## Data Availability

All assemblies provided in this article are available under the NCBI BioProject PRJNA853936: https://www.ncbi.nlm.nih.gov/bioproject/PRJNA853936. All data are provided in full in this paper except for data in Supporting Information: Tables S1–S8, which are available in the figshare repository at https://10.6084/m9.figshare.c.6226566 (Table S1: dataset metadata and assembly metrics. https://doi.org/10.6084/m9.figshare.21251391.v1; Table S2: *B. ceti* and *B. pinnipedialis* pan‐genome annotation. https://doi.org/10.6084/m9.figshare.21251394.v1; Table S3: *B. ceti* pan‐genome KEGG annotation. https://doi.org/10.6084/m9.figshare.21251403.v1; Table S4: *B. pinnipedialis* pan‐genome KEGG annotation. https://doi.org/10.6084/m9.figshare.21251412.v1; Table S5: *B. ceti* virulence factors and resistance genes. https://doi.org/10.6084/m9.figshare.21251406.v1; Table S6: *B. pinnipedialis* virulence factors and resistance genes. https://doi.org/10.6084/m9.figshare.21251409.v1; Table S7: *B. ceti* and *B. pinnipedialis* pseudogenes putative matches against VFDB. https://doi.org/10.6084/m9.figshare.21251397.v1; Table S8: *B. ceti* and *B. pinnipedialis* annotated prophages sequences according to the PHASTER web server. https://doi.org/10.6084/m9.figshare.21251400.v1).
